# Necrology

**Published:** 1899-02

**Authors:** 


### NECROLOGY.

John C. Meyer, Sr., V.S., died at his home, 2230 St. James Avenue, Cincinnati, Ohio, January 16th, of heart-failure, caused by the grippe, from which he had been a sufferer for some time. Deceased was a graduate of Stuttgart and Vienna, class of 1846.

In 1849 he came to the United States, following his profession in Reading, Pa., and Trenton, N. J., going West in 1859, locating at Cincinnati, Ohio, where he enjoyed a lucrative practice until 1893, when, owing to impaired health, he retired from his professional pursuits. He was a member of the American and Ohio Veterinary Medical Associations. Few men have practised veterinary science in our country and left such a distinguished reputation among their people as has Dr. Meyer. His ability as a practitioner was of the highest type; his steadfastness of purpose in daily adding to the worth of his profession to the people; his frequent contributions to veterinary literature, giving excellent advice based on keen observation, and his arrival at sound deductions, were prominent traits in his long and useful life in his much-loved profession. He leaves a widow, four daughters, and one son.

At Lancaster, Pa., January 10, 1899, Henry C. Smith, V.S. Dr. Smith was a non-graduate and practised among the farmers of that county. He had held minor offices of trust in his district. Unfortunate speculations and loss of money caused him to take his own life.

